# The association between *NFKB1 *-94ATTG ins/del and *NFKB1A* 826C/T genetic variations and coronary artery disease risk

**DOI:** 10.22099/mbrc.2018.28261.1302

**Published:** 2018-03

**Authors:** Abbas Seidi, Sina Mirzaahmadi, Khalil Mahmoodi, Mohammad Soleiman-Soltanpour

**Affiliations:** 1 Department of Genetic, Faculty of Basic Sciences, Islamic Azad University, Zanjan Branch, Zanjan, Iran; 2 Department of Cardiology, School of Medicine, Zanjan University of Medical Sciences, Zanjan, Iran; 3 Department of Medical Laboratory Sciences, School of Paramedical Sciences, Zanjan University of Medical Sciences, Zanjan, Iran

**Keywords:** Coronary artery disease, Nuclear factor κB, Polymorphism

## Abstract

Coronary artery disease (CAD) is considered as a chronic inflammatory disease initiated from early childhood. Nuclear factor κB (NF κB) and κB1A (NF κB1A) are the key regulators of inflammatory responses. The *NFKB1* -94ATTG ins/del and *NFKB1A* -826C/T polymorphisms may contribute to the development of CAD. The aim of the present study was to investigate the association of these polymorphisms with the risk of CAD. The study population included 120 patients with angiographically confirmed CAD and 100 matched controls. Genotyping of *NFKB1* -94ATTG ins/del and *NFKB1A* -826C/T polymorphism was performed using PCR-RFLP method. Lipid level was determined by routine colorimetric methods. Statistical analysis was done by SPSS 16 software. Results indicated that the genotypic (P=0.041) and allelic (P=0.009) distribution of the NFKB1-94ATTG ins/del polymorphism was significantly different between the two groups. In the univariate analysis (ins/ins genotype as reference), the del/del genotype (OR=2.88, 95% CI=1.21-6.84, P=0.015) but not ins/del genotype (OR=1.48, 95% CI=0.83-2.64, P=0.191) was significantly associated with the increased risk of CAD. In the multiple binary logistic regression analysis, diabetes, hypertension, smoking, LDL-cholesterol, total cholesterol, HDL-cholesterol and *NFKB1* -94ATTG del/del genotype were identified as significant and independent risk factors for CAD development. The distribution of genotypes and alleles of *NFKB1A* -826C/T polymorphism was not significantly different between the two groups. In conclusion the present study identified *NFKB1* -94ATTG ins/del polymorphism but not *NFKB1A* -826C/T polymorphism as a significant and independent risk factor for development and severity of CAD.

## INTRODUCTION

Coronary artery diseases (CAD) remain the leading causes of morbidity and mortality worldwide. Several lines of evidence have shown that inflammatory process plays crucial roles in the development of CAD. Inflammatory process participates in all stages of atherosclerosis from its initial development to its final progression to thrombotic complications [1]. The nuclear factor κB (NF-κB) is a family of transcription factors that regulate the expression of various genes encoding pro-inflammatory and anti-inflammatory protein. It also regulates the transcription of different genes involved in the pathogenesis of CAD such as pro inflammatory cytokines, chemokines, adhesion molecules, and acute phase reactant proteins [2, 3]. The NF-kB transcription factor family consists of five subunits including, RelA (p65), RelB, c-Rel, NF-κB1 (p50/p105), and NF-κB2 (p52/p100) [4-6]. Different heterodimeric and homodimeric combinations of these subunits were shown to target different genes. The NF-kB1 gene located in chromosome 4 encodes two different subunits; p50 with a DNA binding site derived from C-terminal of p105, and p105 which has no DNA binding activity [7]. The p50/p50 homodimer preferentially stimulates the transcription of the anti-inflammatory cytokine such as IL-10, while suppresses the transcription of pro-inflammatory cytokines like IL-12 and TNF-α. Conversely, the p50/p65 heterodimer induces the transcription of genes with pro-inflammatory property [3-5, 8]. 

A functional polymorphism (-94 ATTG ins/del, rs28362491) located in the promoter region of *NFKB1* is the result of a four nucleotide ATTG insertion/deletion variant which leads to reduced promoter activity and decreased production of p50 subunit [9, 10]. The variant homozygous deletion (del/del) genotype produces lower levels of p50 subunits and has been associated with many inflammatory diseases such as systemic lupus erythematosus, Grave’s disease, ulcerative colitis and cancer [2-5, 11]. The activity of NF-kB is mainly regulated by IκBα, which binds to NF-kB in the cytoplasm of the cells and inhibits its translocation to the nucleus. So, the IκBα suppresses the transcriptional activity of NF-kB by sequestering and degradation of NF-kB in the cytoplasm of the cells [2, 12]. However, under inflammatory conditions such as exposure to lipopolysaccharide, reactive oxygen species (ROS) and tumor necrosis factor alpha (TNF-α), the IκBα is phosphorylated and degraded in the cytoplasm which consequently results in activation of the NF-kB complex [2, 12]. 

The IκBα is encoded by *NFKB1A* gene on chromosome 14 [13]. The -826C/T (rs 2233406) promoter polymorphism of *NFKB1A* has been proposed as a significant risk factor for various inflammatory diseases and cancers with conflicting results [9, 14-16]. The present study was designed to determine the association of *NFKB1*-94 ATTG ins/del and *NFKB1A* -826C/T promoter polymorphisms with the development and severity of CAD in an Iranian subpopulation.

## MATERIALS AND METHODS

**Subjects:** The studied population encompassed 220 subjects including 120 patients (68 male and 52 female) with a diagnosis of CAD confirmed by angiography and 100 matched controls (54 male and 46 female). The mean age of CAD patients and controls were 60.34 ± 12.2 and 58.3 ± 12.6, respectively. The diagnosis of CAD was made by an expert cardiovascular specialist through angiography. The main criteria for inclusion of CAD patients in the study included the presence of 50% or more stenosis in at least one major coronary vessel. Regarding the severity of CAD, all of the CAD patients were categorized in to single, double and triple vessel stenosis based on the number of stenotic vessel showing ≥50% stenosis. Patients showing fewer than 50% stenosis or taking lipid-lowering drugs as well as patients with cardiomyopathy, valvular heart disease, auto immune disorders, inflammatory disease, infectious disease, major organ failures and cancer were excluded from the study. Control subjects were included in the study based on the absence of any personal or family history of CAD or other reasons to suspect CAD. Moreover, control subjects with overt concomitant diseases such as autoimmune disease, infectious disease malignant diseases were excluded. A complete medical history regarding the smoking habits, hypertension, diabetes and family history of heart disease was obtained by questionnaire. Diabetes was defined by fasting blood glucose >126mg/dL and hypertension was defined by systolic blood pressure>140 mmHg and/or diastolic blood pressure >90 mmHg. All of the study subjects participated voluntarily in the study and written informed consent was obtained from all participants. The study was approved by the ethical committee of Zanjan Azad University, Zanjan, Iran. 

**Sample collection: **After 10-12 hours fasting, 7 ml blood was collected in EDTA containing tubes. Then the samples were instantly centrifuged and the separated plasma was stored at -20ºC until biochemical analysis. Also, the cellular fraction was used for DNA extraction.

**Genotyping: **Genomic DNA was extracted from blood leukocytes using a standard salting out method [17]. The NF-kB1 -94 ATTG ins/del and *NFKB1A* -826C/T polymorphisms were genotyped by polymerase chain reaction combined with restriction fragment length polymorphism (PCR-RFLP). The sequence of primers for *NFKB1* -94 ATTG ins/del was as follows: forward primer TGG GCA CAAGTC GTT TAT GA and reverse primer CTG GAG CCG GTA GGG AAG which amplified a 281 bp fragment in the presence of del allele and a 285 bp fragment in the presence of ins allele. Also, the sequence of primers used for *NFKB1A* -826C/T polymorphism was as follows: forward primer GGT CCT TAA GGT CCA ATC G and reverse primer GTT GTG GAT ACC TTG CAC TA which produced a 200 bp fragment. After amplification, a 7 microliter aliquot of *NFKB1* PCR product was digested with 5U *PfIM*I (Van91I) restriction enzyme (Fermentas, Germany) for at least 8 hours at 37 °C. Then the digested product were separated on 3% agarose gel and stained with Sybr green dye. The wild type allele (ins) contain PflMI (Van91I) restriction site and is cleaved in to 240 bp and 45 bp fragments. The mutant allele (del) didn’t contain PflMI restriction site and remained un-cleaved as a 281bp band. Similarly, a 7 microliter aliquot of *NFKB1A* PCR product was digested with 5U *Bfa* I restriction enzyme (Fermentas, Germany) for at least 8 hours at 37 °C. Following electrophoresis on 3% agarose gel, the 200 bp fragment was separated as 180bp and 20 bp band in the presence of -826T allele and as a single 200bp undigested fragment in the presence of -826C allele. 

**Biochemical methods:** Plasma total cholesterol (TC), triglycerides (TG), high density lipoproteine- cholesterol (HDL-C), low density lipoprotein- cholesterol (LDL-C) and fasting glucose levels were measured with commercially available enzyme assay kits (Pars Azmon Co, Tehran, Iran) using Mindray auto-analyzer (BS-200). 

**Statistical analysis**: In descriptive statistics, numerical variables presented as mean ± SD and were compared using Student t- test. Categorical variables were compared with Chi square test or Fisher exact test (in case of small sample size).  The deviation of genotype distribution from the Hardy-Weinberg equilibrium (HWE) was assessed in both patients and controls by Chi-square test. Binary logistic regression analysis was performed to determine the independent association of each covariate with the risk of CAD. The statistical analysis with a P values<0.05 was considered significant. All statistical analysis was performed using SPSS 16 software.

## RESULTS

The clinical and demographic characteristics of the CAD patients and control subjects are presented in Table 1. As indicated, mean age, sex distribution and TG levels were similar between the two groups. However, the distribution of hypertension, smoking status and diabetes were significantly different between the CAD group and control group (Table 1). Also, CAD patients had significantly higher TC, LDL-C levels and lower HDL-C levels than control subjects. 

**Table 1:** Biochemical and demographic characteristics of the CAD patients and the control subjects included in the study

**Variables**	**CAD group**	**Control group**	***P-*** **value**
**Gender**			
Female	52	46	0.785
Male	68	54	
**Diabetes**			
Absent	83	90	
Present	27	10	0.017
**Smoking status**			
Non-smoker	78	89	
Smoker	42	11	<0.001
**Hypertension**			
Absent	95	90	
Present	25	10	0.040
**Age (years)**	60.34 ± 12.2	58.3 ± 12.6	0.225
**TG (mg/dl)**	183.5 ±94.9	170.1 ± 76.9	0.257
**TC (mg/dl)**	193.12 ± 65.8	169.7 ± 48.3	0.003
**HDL-C (mg/dl)**	38.36 ± 11.1	42.6 ± 15.1	0.017
**LDL-C (mg/dl)**	103.7 ± 52.5	85.2 ± 46.4	0.006

TC: total cholesterol, TG: Triglyceride, HDL: High-density lipoprotein, LDL: Low-density lipoprotein, CAD: Coronary artery disease

The genotypes distribution of *NFKB1* -94 ATTG ins/del polymorphism and *NFKBIA* -826C/T polymorphism did not reveal any significant deviation from Hardy Weinberg equilibrium in CAD group and control group (Table 2). The genotype distribution of the *NFKB1* -94ATTG ins/del polymorphism was significantly different between the two groups (χ^2^=6.35, P=0.041). In the univariate analysis, using the ins/ins genotype as a reference, the del/del genotype of *NFKB1* -94 ATTG ins/del polymorphism was significantly associated with the increased risk of CAD (P=0.015). However, the ins/del genotype did not reveal such an association (P=0.191). However, regarding the *NFKBIA* -826C/T polymorphism, no significant difference was seen in the genotype distribution (χ^2^=2.25, P=0.324) of *NFKBIA* -826C/T polymorphism between the two groups. 

**Table 2:** Genotypes distributions of *NFKBIA* -826 C/T and *NFKB1* -94ATTG ins/del polymorphisms in CAD group and control groups

**Polymorphisms**	**CAD group**	**Control Group**	**OR (95% Cl)**	***P*** **-value**
**NF-** **𝜅** **B1 rs28362491**				
**ins/ins**	47	53	Ref	-
**Ins/del**	50	38	1.48 (0.83-2.64)	0.191
**del/del**	23	09	2.88 (1.21-6.84)	0.015
**NF-** **𝜅** **BIA rs 2233406**				
**CC**	62	60		
**CT**	42	32	1.27 (0.71-2.27)	0.46
**TT**	16	08	1.93 (0.77-4.85)	0.183

Moreover, to further confirm the independent association of each risk factor with CAD occurrence, binary logistic regression analysis was done. Results indicated several independent risk factors for CAD development, including del/del genotype of *NFKB1* -94 ATTG ins/del polymorphism, TC, HDL-C, LDL-C, smoking, diabetes and hypertension. However, some other variables such as age, sex, TG and-826 TT and -826CT genotypes of *NFKBIA* -826C/T polymorphism were not identified as significant risk factors for CAD (Table 3). Moreover, we performed a subpopulation analysis to investigate the gene-environmental interaction between *NFKBIA* -826C/T polymorphism and some CAD risk factors such as hypertension, smoking and diabetes. As indicated in Table 4, the NFKBIA -826C/T polymorphism did not increase the risk of CAD in hypertensive, diabetic or smoker subpopulations of CAD patients.

**Table 3:** Multiple binary logistic regression analysis of CAD patients and control subjects

**Covariates**	**OR**	**95% CI**	***P-*** **value**
**Age (years)**	1.03	(0.98-1.05)	0.475
**Sex (M/F)**	0.95	(0.67-1.52)	0.901
**Smoking (%)**	3.83	(1.67-8.68)	0.005
**Triglyceride**	1.005	(1.003-1.011)	0.098
**Total Cholesterol**	1.009	(1.005-1.016)	0.007
**HDL- Cholesterol**	0.957	(0.917-1.021)	0.025
**LDL- Cholesterol**	1.006	(1.001-1.015)	0.011
**Diabetes**	2.12	(0.98-5.32)	0.028
**Hypertension**	2.16	(1.03-4.82)	0.042
**rs 28362491 (del/del vs ins/ins)**	2.37	1.11-5.64	0.021
**rs 28362491 (del/del vs ins/ins +ins/del)**	1.97	1.02- 5.18	0.040
**rs 2233406 (TT vs. CC)**	1.71	0.56-4.23	0.135
**rs 2233406 (CT vs. CC)**	1.32	0.84-2.23	0.364

**Table 4:** Interaction between rs2233406 polymorphism and demographic characteristics of subjects in the risk of coronary artery disease

**Variable**	**NF-** **𝜅** **BIA -826C/T (rs 2233406)**						
	**CC+CT**		**TT**		**OR**	**95% CI **	***P*** **-value**
	**Case**	**Control**	**Case**	**Control**			
**Hypertension**							
No	88	84	7	6	1.11	0.35 -3.45	0.981
Yes	16	8	9	2	2.25	0.39-12.97	0.447
**Smoking**							
No	74	86	4	3	1.55	0.33-7.15	0.706
Yes	30	6	12	5	0.48	0.12- 1.87	0.301
**Diabetes**							
No	87	86	6	4	1.48	0.40 -5.44	0.747
Yes	17	6	10	4	0.88	0.19 -3.90	0.999

In order to investigate the role of *NFKB1* -94 ATTG ins/del polymorphism in determining the severity of CAD, the genotype distribution of *NFKB1* -94 ATTG ins/del polymorphism was compared among patients with one, two or three stenotic vessels. Results indicated that the presence of del allele containing genotypes was significantly more common in patients with three or two stenotic vessels relative to patients with single stenotic vessels [Table 5].

**Table 5:** The association between the number of stenotic coronary vessels and rs28362491 polymorphism

**94ATTG ins/del**	**1 Stenotic**	**2 Stenotic**	**3 Stenotic**	**2 vs. 1 Stenotic Vessel**	**3 vs. 1 Stenotic Vessel**
**Genotypes**	**Vessel **	**Vessel **	**Vessel**	***P*** **-value**	***P*** **-value**
	**N=44**	**N=49**	**N=27**		
**ins/ins**	27	13	7	Ref	Ref
**ins/del **	10	30	10	0.001	0.035
**del/del **	7	6	10	0.505	0.011
**ins/del + del/del **	17	36	20	0.009	0.006

## DISCUSSION

Coronary artery disease (CAD) is currently considered as a progressive inflammatory disease initiated during early childhood. Inflammatory cytokines and their signaling pathways play a vital role in the development of CAD. The *NFKB1* and *NFKBIA* genes display pivotal roles in the regulation of inflammatory responses and genetic variations in these genes has been documented in the several pathologies [2, 4, 5, 12]. The *NFKB1* -94 ATTG ins/del polymorphism and *NFKBIA* -826C/T polymorphism was shown to alter the activation of NFKB pathway and may contribute to the development of CAD [2, 9, 10, 14, 18]. 

The present study investigated the association between promoter polymorphism in *NFKB1* and *NFKBIA* genes and CAD risk in an Iranian population. The main findings were that the distribution of *NFKB1* del/del genotype was significantly higher in CAD cases compared to controls. Carriers of *NFKB1* del/del genotype had 2.88 fold increased risk for development of CAD compared with carriers of ins/ins genotype (P=0.015). Also, the severity of CAD as determined by the number of stenotic vessels was significantly associated with the mutant del allele of NFKB1 -94 ATTG ins/del polymorphism. However, NFKBIA -826C/T polymorphism did not reveal any significant association with the development and severity of CAD. 

Our findings were in agreement with some previously published studies that reported significant association between *NFKB1* -94 ATTG ins/del polymorphism and CAD occurrence [9, 18-20]. Lai et al. studied the prevalence of *NFKB1* -94 ATTG ins/del and *NFKBIA* -826C/T polymorphism in a case control study and concluded that *NFKB1* -94 ATTG ins/del polymorphism was correlated with the increased risk of CAD in a recessive genetic model [9]. Luo et al. investigated the prevalence of *NFKB1* -94 ATTG ins/del polymorphism in CAD patients and control subjects and indicated that frequency of the del/del (DD) genotype and del (D) allele was significantly higher in CAD patients than that of in control subjects [20]. Arslan et al. studied the association between CAD and *NFKB1* 94 I/D polymorphism in a Turkish population and reported a significant association between this common polymorphism and CAD occurrence [18]. By contrast, the study by Oner et al., which investigated the prevalence of *NFKB1* -94 ATTG ins/del polymorphism in a group of CAD patients and healthy control subjects, no significant association was seen between this common polymorphism and CAD occurrence in either recessive, dominant or allelic genetic models [21]. Also, Boccardi et al. in a study of Italian population reported that *NFKB1* -94 ATTG ins/del polymorphism plays a protective role against CAD development and showed that carriage of *NFKB1*-94 ATTG ins/del gene variant confers a lower susceptibility to myocardial infarction incidence which was inconsistent with the present study [22]. In current study a significant association was seen between *NFKB1* -94 ATTG ins/del gene variant and the number of stenotic vessels, indicating that this common polymorphism may be involved in determining the severity of CAD. In accordance with this finding, a study by Luo et al. found a positive association between *NFKB1* -94 ATTG del/del homozygous mutant genotype and severity of CAD [20].

*NFKB1* gene encodes p50 subunit of NF-κB that is utilized for production of p50-p50 homodimer. This homodimer blocks the transcription of anti-inflammatory cytokines such as IL-10 and stimulate the transcription of pro-inflammatory cytokine including IL-6. The *NFKB1*-94 ins/del ATTG promoter polymorphism results in partial depletion of p50 subunit and p50-p50 homodimer and thereby exacerbates inflammatory state [2].

Moreover, according to binary logistic regression analysis, the association between the NF-𝜅B1 del/del genotype and increased risk of CAD remained significant after adjustment for major risk factors, indicating that the *NFKB1* -94 ins/del ATTG polymorphism can affect CAD risk mainly through inflammatory pathways which is independent of established risk factors. This finding is consistent with previous studies [2]. 

Regarding the role of *NFKBIA* -826C/T polymorphism in CAD pathogenesis conflicting results have been reported. In agreement with the present study Lai et al., found no significant association between *NFKBIA* -826C/T polymorphism and CAD development [9]. However, in a study by Özbilüm et al. it was reported that the TT genotype and T allele frequency of *NFKBIA* -826C/T in the CAD group was significantly higher than that of the control group [14]. Several mechanisms may be involved in the inconsistency of association studies. Numerous factors including the sample size, sample selection criteria, different genetic and ethnic background of studied populations, misclassification of phenotypes, interactions between gene-gene and gene- environment and other unknown factors may be attributable for the discrepant results of association studies [23].

The present study bears a number of limitations including (i) the levels of inflammatory cytokines such as IL6 and TNF-α were not determined (ii) this is a single-center study representing a relatively small numbers of patients. In conclusion our preliminary study indicated that *NFKB1* -94 ATTG ins/del polymorphism but not *NFKBIA* -826C/T polymorphism increased the risk and severity of CAD in an Iranian population. However, this preliminary results need to be replicated in future studies with large sample size.

## Conflict of Interest:

There is no conflict of interests to be declared regarding the publication of this paper. 
